# Effect of murine exposure to gamma rays on the interplay between Th1 and Th2 lymphocytes

**DOI:** 10.3389/fphar.2015.00074

**Published:** 2015-04-09

**Authors:** Amany A. Ghazy, Salma Y. Abu El-Nazar, Hossam E. Ghoneim, Abdul-Rahman M. Taha, Amira M. Abouelella

**Affiliations:** ^1^Department of Immunology, Medical Research Institute, Alexandria UniversityAlexandria, Egypt; ^2^Department of Medical Biophysics, Medical Research Institute, Alexandria UniversityAlexandria, Egypt; ^3^Department of Radiation Biology, National Centre for Radiation Research and Technology, Atomic Energy AuthorityCairo, Egypt

**Keywords:** gamma radiation, lymphoproliferative response, polyclonal mitogens, interleukin-12 (IL-12), IL-10, Th1/Th2 response

## Abstract

Gamma radiation radiotherapy is one of the widely used treatments for cancer. There is an accumulating evidence that adaptive immunity is significantly contributes to the efficacy of radiotherapy. This study is carried out to investigate the effect of gamma rays on the interplay between Th1/Th2 response, splenocyte lymphoproliferative response to polyclonal mitogenic activators and lymphocytic capacity to produce IL-12 and IL-10 in mice. Results showed that exposure of intact spleens to different doses of γ-rays (5, 10, 20 Gy) caused spontaneous and dose-dependent immune stimulation manifested by enhanced cell proliferation and elevated IL-12 production with decreased IL-10 release (i.e., Th1 bias). While exposure of splenocytes suspension to different doses of γ-rays (5, 10, 20 Gy) showed activation in splenocytes stimulated by PWM at 5 Gy then a state of conventional immune suppression that is characterized by being dose-dependent and is manifested by decreased cell proliferation and IL-12 release accompanied by increase in IL-10 production (i.e., Th2 bias). In addition, we investigated the exposure of whole murine bodies to different doses of γ-rays and found that the exposure to low dose γ-rays (0.2 Gy) caused a state of immune stimulation terminated by a remarkable tendency for immune suppression. Exposure to 5 or 10 Gy of γ-rays resulted in a state of immune stimulation (Th1 bias), but exposure to 20 Gy showed a standard state of immune suppression (Th2 bias). The results indicated that apparently we can control the immune response by controlling the dose of γ-rays.

## Introduction

Ionizing radiation (IR) is the mainstay of cancer therapy (Shen et al., 2014). There are several forms of IR, either particulate such as neutrons, α and β-particles or electromagnetic waves as gamma (γ) and x-rays. Gamma and x-rays easily penetrate body tissues and deposit their energy deep in the body (Mettler and Voelz, 2002).

The effect of IR on healthy individuals depends on the dose amount and rate of radiation (Park et al., 2014). Acute exposure to high doses of radiation rapidly leads to major injuries to the immune system and gastrointestinal tract (Moroni et al., 2013). The deleterious effects of radiation are considered to result mainly from direct induction of DNA damage, apoptosis, necrosis, genomic instability, changes in the microenvironment of the major constituents including protein structure and transformation of cells into tumor cells (Abouelella et al., 2007; Mirzayans et al., 2013; Moroni et al., 2013; Park et al., 2014). Low doses of ionized radiation may induce effects that could not be manifested soon after the exposure. Thus, there is no threshold dose and radiation absorption may increase the risk for expression of non-threshold effects in both irradiated individuals and their progeny (Georgieva, 2006).

Cells of the immune system, like most radiosensitive cells, are vulnerable to radiation (Park et al., 2014). Lymphocytes are highly susceptible to DNA damage-induced apoptosis, a suicide response adapted to their high potential for mutation and clonal expansion (Faraj et al., 2010). Radiation exposure induces apoptosis in mature T and B lymphocytes and lethal damage in bone marrow stem cell precursors of monocytes and granulocytes (Park et al., 2014).

T helper (Th) cells are divided into two types, depending on the secreted-cytokine patterns. Th1 cells secrete interleukin-2 (IL-2), interferon-γ (IFN-γ), and IL-12. They activate macrophage and serve as mediators of cell-mediated immune responses, such as delayed-type hypersensitivity (DTH), and promote tumoricidal activity. In contrast, Th2 cells secrete IL-4, IL-5, IL-6, and IL-10. They induce IgE- and eosinophil-mediated reactions and promote humoral immunity (Aruga et al., 1997). Th1 and Th2 cells reciprocally inhibit the growth and function of the other cell type (Han et al., 2002). Irradiation causes alteration in cytokine release from different cell types and shift in immune responses (Noorizadeh et al., 2004).

Radiation can exert various immunomodulatory effects including induction of the expression of cytokines, chemokines, and release of inflammatory mediators (Kaur and Asea, 2012). The difference in the radio-sensitivity of the cells that are involved in immune responses is well-known, yet little is known about the regulation of the cytokine release by irradiated cells.

Yoshiki et al. (2012) have demonstrated that IR has the potential of interrupting the balance between Th1/Th2 cell responses; an effect that is closely associated with the reduction of Th1 cytokine expression and increase in Th2 cytokines’ levels. This radiation induced alteration in Th1/Th2 balance is profoundly related to the modulation of cytokine-mediated transcriptional factors and signaling pathways. They proposed that the role of UVB-mediated immunosuppression is the inhibition of self-destruction against external stresses.

An understanding of the biochemical and molecular mechanisms in pathways evoked by radiation, particularly the immunosuppressive mechanism of gamma irradiation, can lead to the development of more efficient radiotherapies with beneficial biological and immunological consequences. Therefore, we examined the effects of gamma irradiation on the regulation of cytokine release (IL-12 and IL-10) as indicators for Th1 and Th2 response, respectively, and the splenic lymphoproliferative response to different polyclonal mitogenic activators.

## Materials and Methods

### Experimental Animals

This study was conducted on 84 male inbreed C57BI/6j mice aging 6–8 weeks and weighing 20–25 g each. The animals were purchased from Theodor Bilharz Research Institute, Cairo, Egypt. This study was approved by the Ethics Committee of Medical Research Institute, Alexandria University. All experiments conform to the regulatory standards of Medical Research Institute, Alexandria University. All reagents used in the study were supplied by Sigma-Aldrich Chemical (St. Louis, MO, USA) unless otherwise stated.

### Gamma Irradiation

Mice were divided into two main groups; the first group was composed of 63 mice. They were subdivided into three subgroups (21 mice each) according to the dose of given γ-radiation. The process of radiation was carried out at National Centre for Radiation Research and Technology (NCRRT), Atomic Energy Authority, Cairo, Egypt. This was employed according to the method adopted by Pecaut et al. (2007) with certain modifications. The dose of γ-radiation was estimated as a function of both distances between source and mice as well as the intensity of irradiation. According to these calculations, mice were exposed to γ-radiation at doses of 0.2, 5, 10, and 20 Gy from ^137^Cs γ-irradiator (Gamma cell-40) was provided by the NCRRT, Cairo, Egypt, manufactured by the Atomic Energy of Canada. In addition to the total body irradiation (TBI), direct irradiation of intact spleens and isolated splenocyte suspensions (at a concentration of 10 × 10^6^) was performed to test the direct effect of irradiation on our study parameters. The second group was composed of 21 age and sex-matched non γ- irradiated mice as negative control. Animals were sacrificed at variable durations (seven mice each time) after TBI to test the effect of recovery time after irradiation (represented 0, 3, and 7 days) on the study parameters.

### Preparation of Splenocyte Suspension

Spleens were collected separately into sterile plastic petri-dishes (7 cm × 1.5 cm) containing 50 mM phosphate buffer saline (PBS) pH 7.2. Splenocytes were prepared individually from spleens excised from all mice under the study, to prepare sufficient amounts of single cell suspensions, according to the method originally employed by Coligan et al. (1994) under strictly aseptic conditions. Single cell suspension was obtained from each preparation by simple sedimentation to get rid of splenic tissue debris and large cell aggregates. Contaminating RBCs were lysed out by suspending cell pellets in 5 ml sterile ammonium chloride solution (0.83%) for 5 min. Splenocytes were washed twice with 50 mM PBS pH 7.2 and finally suspended in tissue culture media composed of RPMI-1640 (Sigma Aldrich, USA) supplemented with L-glutamine, 10% heat inactivated fetal calf serum, penicillin (100 IU/ml), and streptomycin (100 μg/ml). Single cell suspensions were subsequently employed in tissue cultures.

### Cell Viability Testing

Prepared splenocytes were tested by trypan blue dye exclusion test (Castro-Concha et al., 2005). Cells were then counted using hemocytometer and counts were adjusted at 2 × 10^6^ cells/ml. Volumes of 10 μl of each cell suspension were gently mixed with equal volumes of 0.2% preparation of trypan blue dye and left for 2–5 min at room temperature. Aliquots were examined microscopically in a hemocytometer. Viable cells are characterized by unstained cytoplasm and shiny boundaries whereas non-viable cells have blue cytoplasm and undefined boundaries. The percentage of the viable cells was estimated according to the following formula: % viability = (no. of viable cells/total cell number) × 100.

### Assessment of Cell Proliferation by MTT Assay

The proliferative functions of isolated splenic lymphocytes were monitored by the *in vitro* mitogenic polyclonal activation using two different plant lectins as mitogens; concanavalin A (ConA) and pokeweed (PWM) adopting the standard protocol of Bieback et al. (2003) with minor modifications. For assessment of the state of lymphocytes proliferation following ConA and PWM, the tetrazolium compound MTT (3-(4,5-dimethylthiazol-2-yl)-2,5-diphenyltetrazolium bromide) was added for cultured cells. MTT is reduced by metabolically active cells to insoluble purple formazan dye crystals. Detergent is then added, destructing cell membranes and solubilizing the crystals so that the absorbance can be read using a spectrophotometer. The rate of tetrazolium reduction is directly proportional to the rate of cell proliferation. Thus ConA and PWM responsive splenocytes will have higher absorbance while weakly activated and non-responsive splenocytes will demonstrate lower optical densities reflecting only their basal metabolic state (Riss and Moravec, 1992).

Volumes of 100 μl of supplemented RPMI-1640 tissue culture medium were dispensed into rows of nine wells within 96 wells flat-bottomed micro titer tissue culture plates (Greiner bio-one, Germany). Volumes of 100 μl of splenic lymphocytes suspension corresponding to each of the control and γ-irradiated mice were dispensed in triplicates at final concentration of 2 × 10^6^ cells/ml. Then two groups of wells were pulsed separately with 2 μl of ConA or PWM at a final concentration of 10 μl/ml, respectively. The remaining group was left without mitogen reflecting the basal metabolic activity of the respective splenocyte suspension. The plate was incubated at 37∘C in humidified CO_2_ incubator (5% CO_2_ and 95% O_2_) for 2 days. Then, 10 μl of MTT reagent (Cayman chemical company, Germany) was added to all wells and mixed gently and the plate was incubated for 3–4 h. At the end of incubation period formazan deposits produced within the cells so 100 μl of the ready to use crystal-dissolving solution was added to each well-producing homogenous dark blue to purple solution with different intensities. Finally the absorbance of each well was measured at 570 nm using a microplate reader (Bio-Rad, USA). The average values from triplicate readings of ConA and PWM stimulated versus un-stimulated wells were determined and used to calculate the stimulation index (SI; Singh et al., 2012) as follow: SI = mean of absorbance values of each mitogen stimulated wells/mean of absorbance values of un-stimulated wells.

### Cytokine Production and Assessment of T Helper (Th) Bias

The *in vitro* capability of splenocytes to release IL-10 and IL-12, representative for Th2 and Th1 cells respectively, was evaluated by using short term culture. This was monitored either spontaneously or in the presence of ConA or PWM mitogens and was taken as a monitor for assessment of Th bias following exposure to gamma radiation. Details of the tissue culture protocol were adopted from the standard method developed by Davis (1995). In brief, volumes of 1 ml of splenocyte suspension (2 × 10^6^ cells/ml of supplemented RPMI-1640) corresponding to each of the control and γ-irradiated mice were dispensed in triplicates into 24 wells flat-bottomed micro titer tissue culture plates (Nunclon, Denmark). One group of wells was left without mitogen while the other two were pulsed separately with 10 μl of either ConA or PWM at a final concentration of 10 μl/ml, respectively. The plate was then incubated at 37∘C in humidified CO_2_ incubator (5% CO_2_ and 95% O_2_) for 2 days. At the end of the culture period, the content of each well was collected and centrifuged at 1800 rpm for 10 min at room temperature. Supernatant s were collected and stored at -80∘C until used for measurement of released cytokines.

### Measurement of IL-10 and IL-12

IL-10 and IL-12 cytokines were assayed in culture supernatants corresponding to all mice under the study. This was done by enzyme linked immunosorbant assay (ELISA) using kit commercially available from Ray Biotech (Castilho et al., 2010).

### Statistical Analysis

The data were analyzed using statistical package for social science (SPSS) program version 17.7. Student’s *t*-test was used to compare between the different groups. Paired *t*-test was used for comparison applied to the same group. To avoid alpha error, the level of significance was adjusted at 0.05 divided by the number of comparisons.

## Results

### Effect of γ-Rays Exposure on Cell Proliferation and Cytokine Production after Irradiation of Intact Spleens

Following γ-rays of intact spleens with different doses of γ-rays, irradiated cells were immediately employed in tissue culture (without recovery durations). Results of cell proliferation as well as cytokine release are expressed as O.D. and pg/ml, respectively.

**FIGURE 1:**
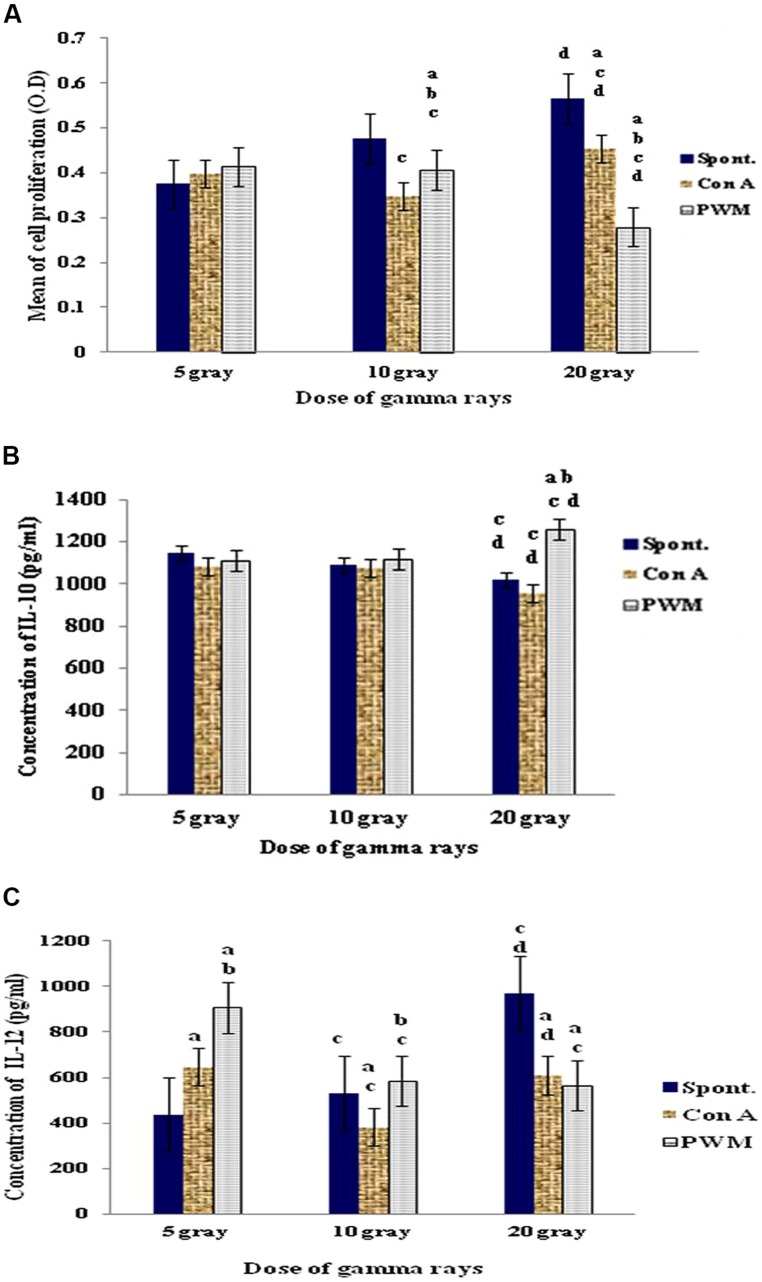
**Diagrammatic representation for comparison between **(A)** cell proliferation (O.D.), **(B)** IL-10 (pg/ml), and **(C)** IL-12 (pg/ml) release either spontaneously or with *in vitro* stimulation with Con A or PWM after irradiation of intact spleens**. ^a^Level of significance between spontaneously and *in vitro* mitogen induced data, ^b^level of significance between Con A and PWM-induced data, ^c^level of significance between data of 5 Gy versus 10 and 20 Gy, ^d^level of significance between data of 10 and 20 Gy.

Irradiation of intact spleens with 5 Gy γ-rays did not show any significant change in basal and mitogen-induced cells proliferation. A dose of 10 Gy γ-rays caused a significant decrease in mitogen-induced cell proliferation as compared to their basal results. In addition, PWM-induced proliferation was significantly higher than that induced by ConA. Use of 20 Gy γ-rays caused significant increase in ConA-induced cell proliferation although PWM-induced proliferation was significantly suppressed when compared to either spontaneous or ConA-induced cell proliferation (**Figure 1**).

Regarding IL-10 release, dose-dependant statistical comparison did not show significant changes between the doses of 5 and 10 Gy γ-rays. However, there was a significant suppression in basal and ConA-induced IL-10 release after exposure of intact spleen to 20 Gy γ-rays (**Figure 1**).

These results showed that exposure of intact spleens to different doses of γ-rays (5, 10, and 20 Gy) caused spontaneous and dose-dependent immune stimulation manifested by enhanced cell proliferation and elevated IL-12 production with decreased IL-10 release (i.e., Th1 bias).

### Effect of γ-Rays Exposure on Cell Proliferation and Cytokine Production after Irradiation of Splenocytes’ Suspensions

Following exposure of splenocytes’ suspensions to different doses of γ-rays, irradiated cells were immediately employed in tissue culture (without recovery durations). Results of cell proliferation and cytokine release expressed as O.D. and pg/ml, respectively.

**FIGURE 2:**
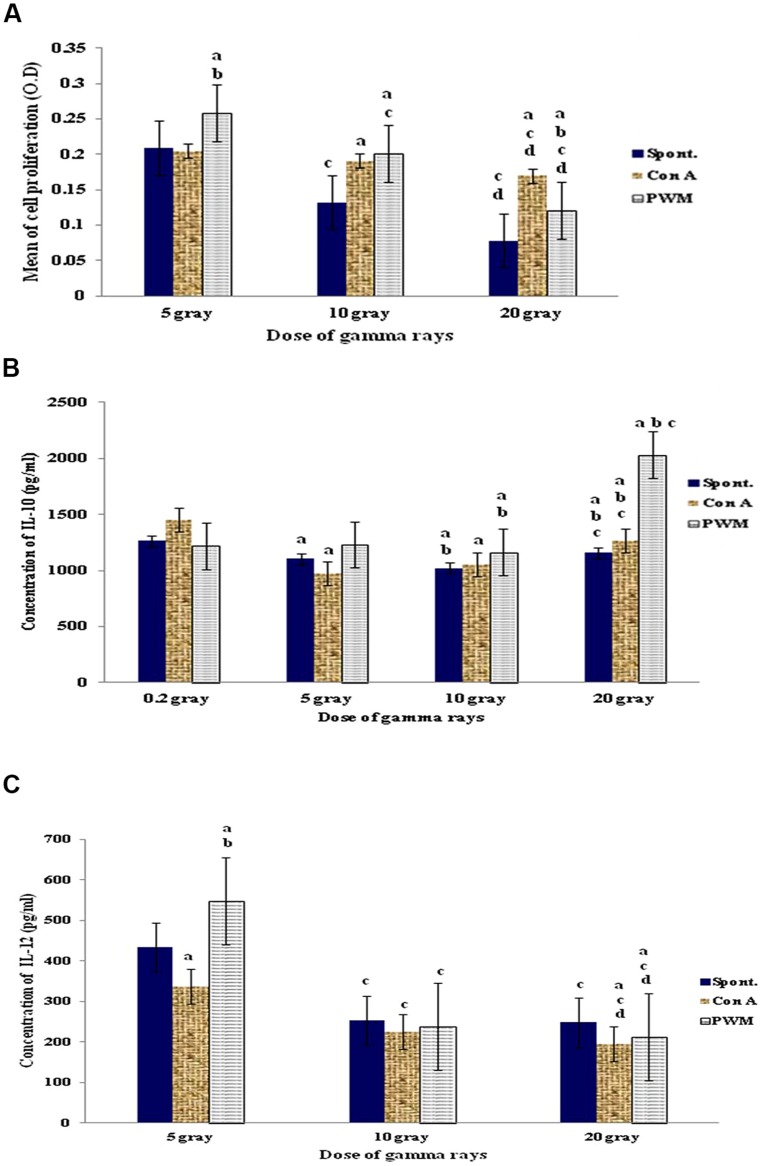
**Diagrammatic representation for comparison between **(A)** cell proliferation (O.D.), **(B)** IL-10 (pg/ml), and **(C)** IL-12 (pg/ml) release either spontaneously or with *in vitro* stimulation with Con A or PWM after irradiation of splenocytes’ suspensions**. ^a^Level of significance between spontaneously and *in vitro* mitogen induced data, ^b^level of significance between Con A and PWM-induced data, ^c^level of significance between data of 5 Gy versus 10 and 20 Gy, ^d^level of significance between data of 10 and 20 Gy.

Statistical analysis of results obtained after irradiation with 5 Gy of γ-irradiation revealed that cell proliferation was significantly enhanced after *in vitro* PWM stimulation. The use of 10 or 20 Gy dose of γ-irradiation caused a significant enhancement in mitogen-induced relative to basal cell proliferation. But there was a significant suppression in spontaneous and mitogen-induced cell proliferation with increasing the dose of γ-rays (**Figure 2A**).

Regarding IL-10 release, exposure to 5 Gy γ-rays did not show any significant variations between basal and mitogen-induced IL-10 release. Exposure to 10 Gy γ-rays caused a significant elevation in IL-10 levels in basal and PWM-induced lymphocytes, while ConA-induced IL-10 release was significantly suppressed. A dose of 20 Gy γ-rays showed a significant elevation of PWM-induced IL-10 release when compared to spontaneous or ConA-induced release (**Figure 2B**).

Regarding IL-12 release, exposure to 5 Gy γ-rays showed increase in PWM-induced IL-12 release when compared to either spontaneous or Con-A-induced release. Increasing the dose of irradiation to 10 then 20 Gy γ-rays caused significant suppression of IL-12 release in all groups of cells. So there was a significant gradual and dose-dependant suppression of IL-12 release either spontaneously or following *in vitro* induction with PWM or ConA (**Figure 2C**).

Thus there is a state of conventional immune suppression that is characterized by being dose-dependent and is manifested by decreased cell proliferation and IL-12 release accompanied by increase in IL-10 production (i.e., Th2 bias).

### Effect of γ-Rays Exposure on Cell Proliferation and Cytokine Production after Whole Body Irradiation

Following exposure of murine whole body to variable doses of γ-rays, mice were left to recover for 0, 3, and 7 days before being sacrificed and dissected in parallel to control non-irradiated mice. Splenocytes were prepared and maintained in short term tissue cultures (for 2 days) either in presence or absence of ConA and PWM (as specific T and B lymphocyte mitogen, respectively). Results are statistically analyzed and expressed in terms of either recovery time or radiation doses.

#### Expression of Cell Proliferation and Cytokine Production as a Function of Recovery Time

**FIGURE 3:**
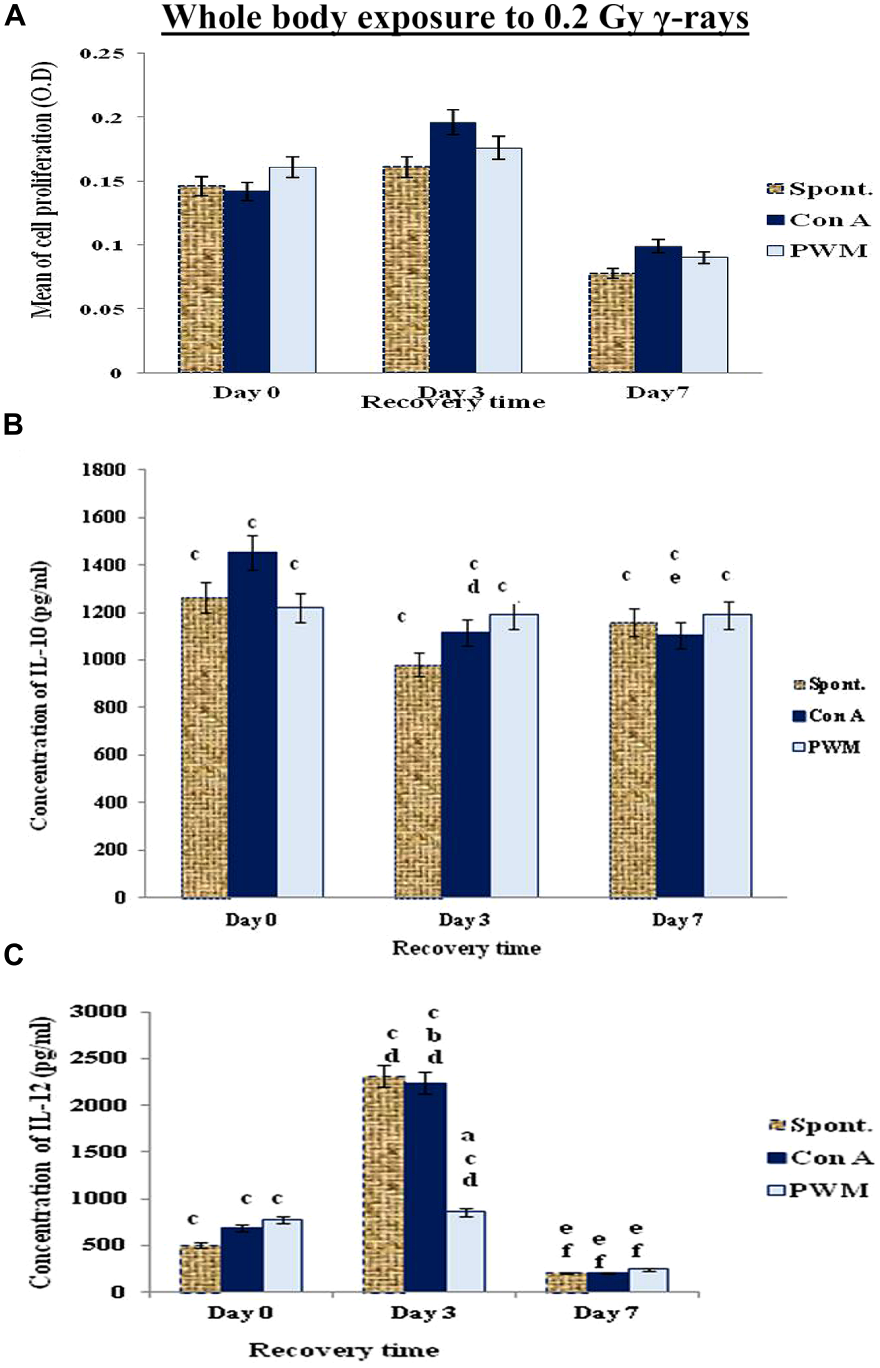
**Diagrammatic representation for comparison between **(A)** cell proliferation (O.D.), **(B)** IL-10 (pg/ml), and **(C)** IL-12 (pg/ml) release either spontaneously or after *in vitro* stimulation with Con A or PWM after whole body exposure to 0.2 Gy γ-rays**. ^a^Level of significance between spontaneously and *in vitro* mitogen induced data, ^b^level of significance between Con A and PWM-induced data, ^c^level of significance between γ-radiation and control data, ^d^level of significance between 0 time and 3 days recovery, ^e^level of significance between 0 time and 7 days recovery, ^f^level of significance between 3 and 7 days recovery.

The data collected at 0 and 3 days recovery after whole bodies’ exposure to low dose γ-rays (0.2 Gy) showed a time-related elevation in cell proliferation and IL-12 release. This was followed by a reduction at 7 days recovery. Although there was a pan decrease in spontaneous and mitogen-induced IL-10 release at 0, 3, and 7 days recovery time relative to their normal, non-irradiated partners. These results indicate a state of immune stimulation following exposure to low dose γ-rays terminated by a remarkable tendency for immune suppression (**Figure 3**).

**FIGURE 4:**
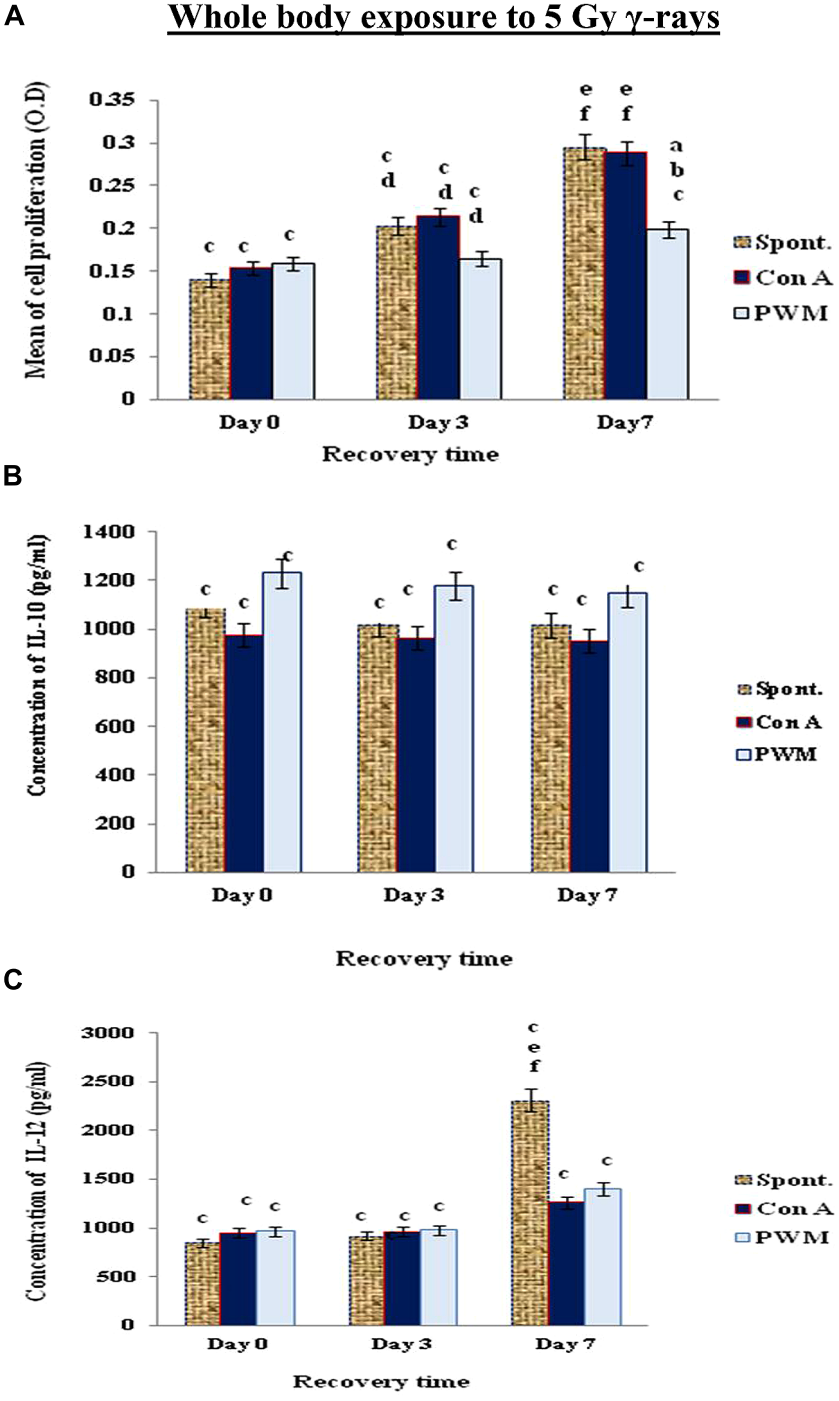
**Diagrammatic representation for comparison between **(A)** cell proliferation (O.D.), **(B)** IL-10 (pg/ml), and **(C)** IL-12 (pg/ml) release either spontaneously or after *in vitro* stimulation with Con A or PWM after whole body exposure to 5 Gy γ-rays**. ^a^Level of significance between spontaneously and *in vitro* mitogen induced data, ^b^level of significance between Con A and PWM-induced data, ^c^level of significance between γ-radiation and control data, ^d^level of significance between 0 time and 3 days recovery, ^e^level of significance between 0 time and 7 days recovery, ^f^level of significance between 3 and 7 days recovery.

When mice exposed to 5 Gy γ-rays, there was a time-dependant enhancement of cell proliferation and IL-12 till day 7 recovery, while IL-10 release was suppressed without remarkable tendency for recovery even after 7 days. These results indicate that a classical state of immune stimulation (Th1 bias) has developed when mice exposed to 5 Gy γ-rays (**Figure 4**).

**FIGURE 5:**
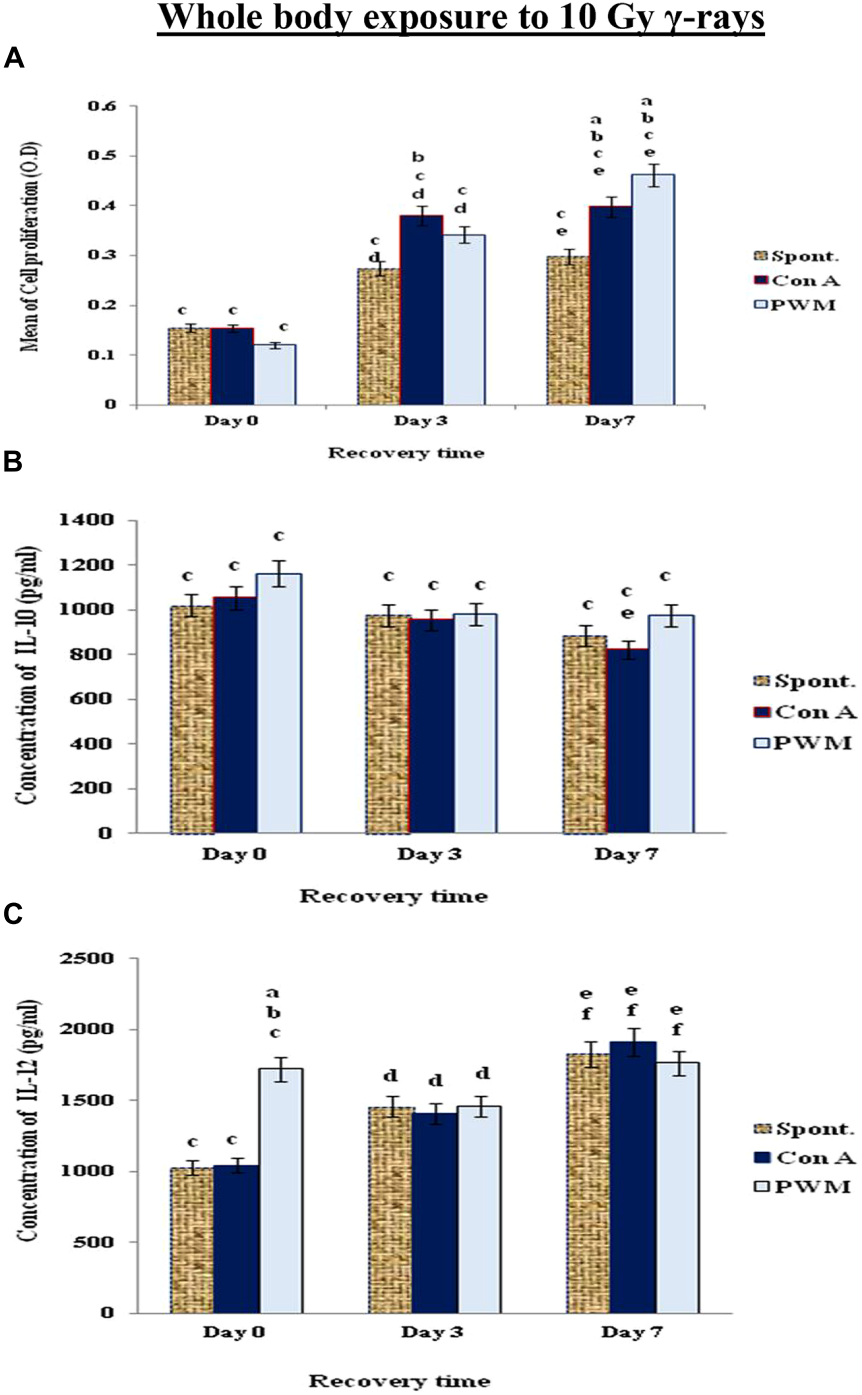
**Diagrammatic representation for comparison between **(A)** cell proliferation (O.D.), **(B)** IL-10 (pg/ml) and **(C)** IL-12 (pg/ml) release either spontaneously or after *in vitro* stimulation with Con A or PWM after whole body exposure to 10 Gy γ-rays**. ^a^Level of significance between spontaneously and *in vitro* mitogen induced data, ^b^level of significance between Con A and PWM-induced data, ^c^level of significance between γ-radiation and control data, ^d^level of significance between 0 time and 3 days recovery, ^e^level of significance between 0 time and 7 days recovery, ^f^level of significance between 3 and 7 days recovery.

When mice exposed to 10 Gy of γ-rays, there was a significant and time-dependant enhancement of cell proliferation while IL-12 was significantly elevated at 0 time only. IL-10 release was suppressed indicating a state of non-classical immune stimulation (Th1 bias; **Figure 5**).

**FIGURE 6:**
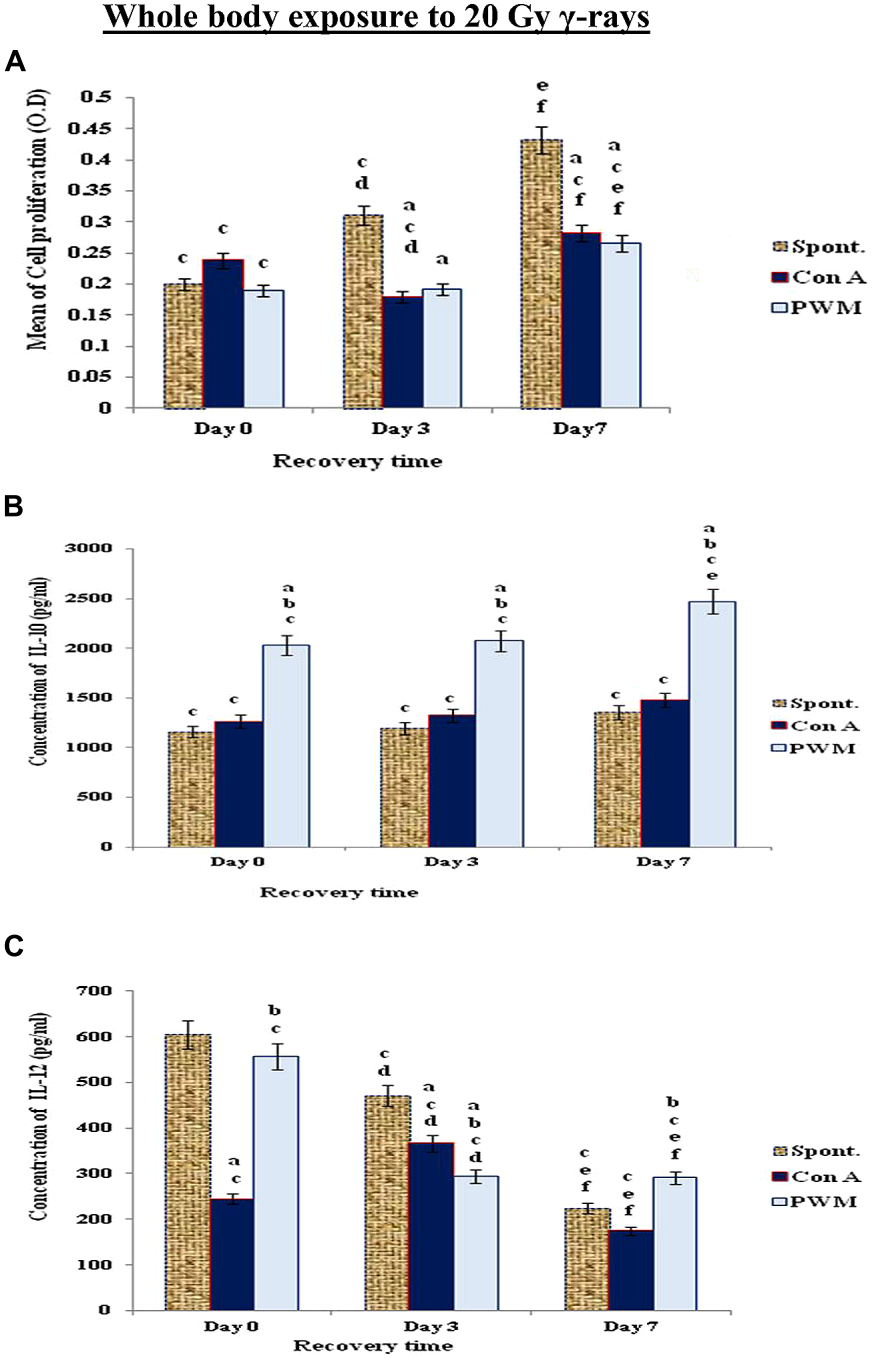
**Diagrammatic representation for comparison between **(A)** cell proliferation (O.D.), **(B)** IL-10 (pg/ml), and **(C)** IL-12 (pg/ml) release either spontaneously or after *in vitro* stimulation with Con A or PWM after whole body exposure to 20 Gy γ-rays**. ^a^Level of significance between spontaneously and *in vitro* mitogen induced data, ^b^level of significance between Con A and PWM-induced data, ^c^level of significance between γ-radiation and control data, ^d^level of significance between 0 time and 3 days recovery, ^e^level of significance between 0 time and 7 days recovery, ^f^level of significance between 3 and 7 days recovery.

When mice exposed to the highest dose γ-rays (20 Gy), there was a remarkable and time-dependent stimulation of cell proliferation associated with significant decrease in IL-12 release and enhancement in IL-10 release indicating that mice exposed to higher doses of γ-rays suffered a standard state of immune suppression (Th2 bias; **Figure 6**).

#### Expression of Cell Proliferation and Cytokine Production as a Function of γ-Rays Dose

The results of the present study were analyzed as a function of γ-rays dose-dependence at fixed time of recovery. We found that exposure to low dose γ-rays (0.2 Gy) caused a state of immune stimulation terminated by a remarkable tendency for immune suppression. Exposure to 5 or 10 Gy of γ-rays resulted in a state of immune stimulation (Th1 bias), but exposure to 20 Gy showed a standard state of immune suppression (Th2 bias). These results indicated that we can control the immune response by controlling the dose of γ-rays ( **Table 1**).

**Table 1:** **The impact of different doses of γ-irradiation on Th1/Th2**.

Dose of gamma rays	Effect
Exposure to 0.2 Gy of γ-rays	State of immune stimulation terminated by a remarkable tendency for immune suppression
Exposure to 5 or 10 Gy of γ-rays	State of immune stimulation (Th1 bias)
Exposure to 20 Gy	Standard state of immune suppression (Th2 bias)

**FIGURE 7:**
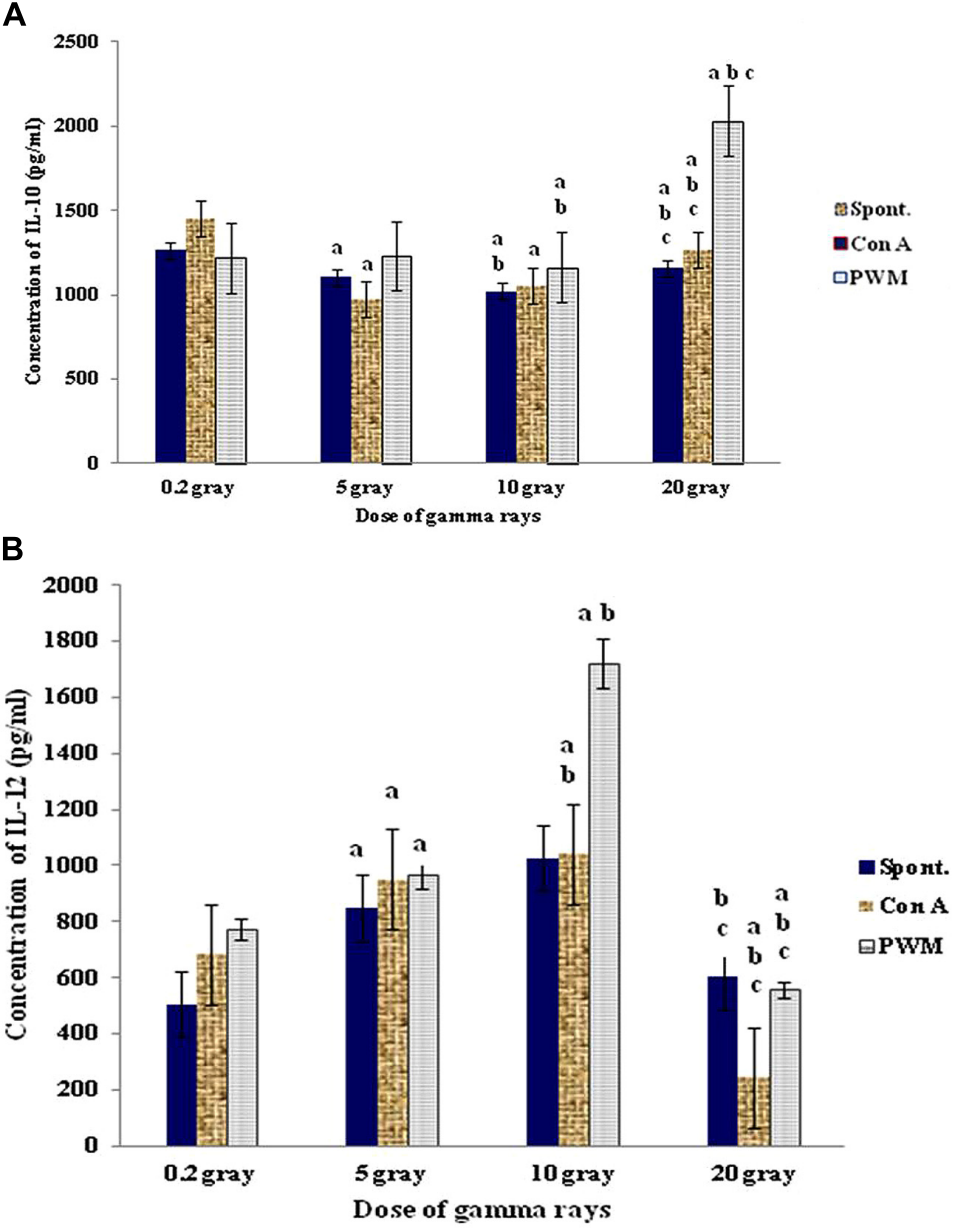
**Concentration of IL-10 (A) and IL-12 (B) release either spontaneously or with *in vitro* stimulation with Con A or PWM at 0 time recovery**. ^a^Level of significance between low doses (0.2 Gy) and higher doses (5,10, 20 Gy) of γ-radiation, ^b^level of significance between 5 Gy and higher doses (10, 20 Gy) γ-radiation, ^c^level of significance 10 and 20 Gy γ-rays.

At 0 time recovery (immediately after exposure), the 20 Gy dose gave the highest degree of cell proliferation although IL-12 was gradually increased by increasing the dose till 10 Gy then showed remarkable decrease with 20 Gy dose of γ-rays. This was accompanied by significant decrease in IL-10 release capacity till 10 Gy then marked elevation at dose of 20 Gy especially with PWM stimulation. These results indicate that doses till 10 Gy were giving a state of immune stimulation (Th1 bias) while the dose of 20 Gy was typically suppressive (Th2 bias; **Figure 7**).

**FIGURE 8:**
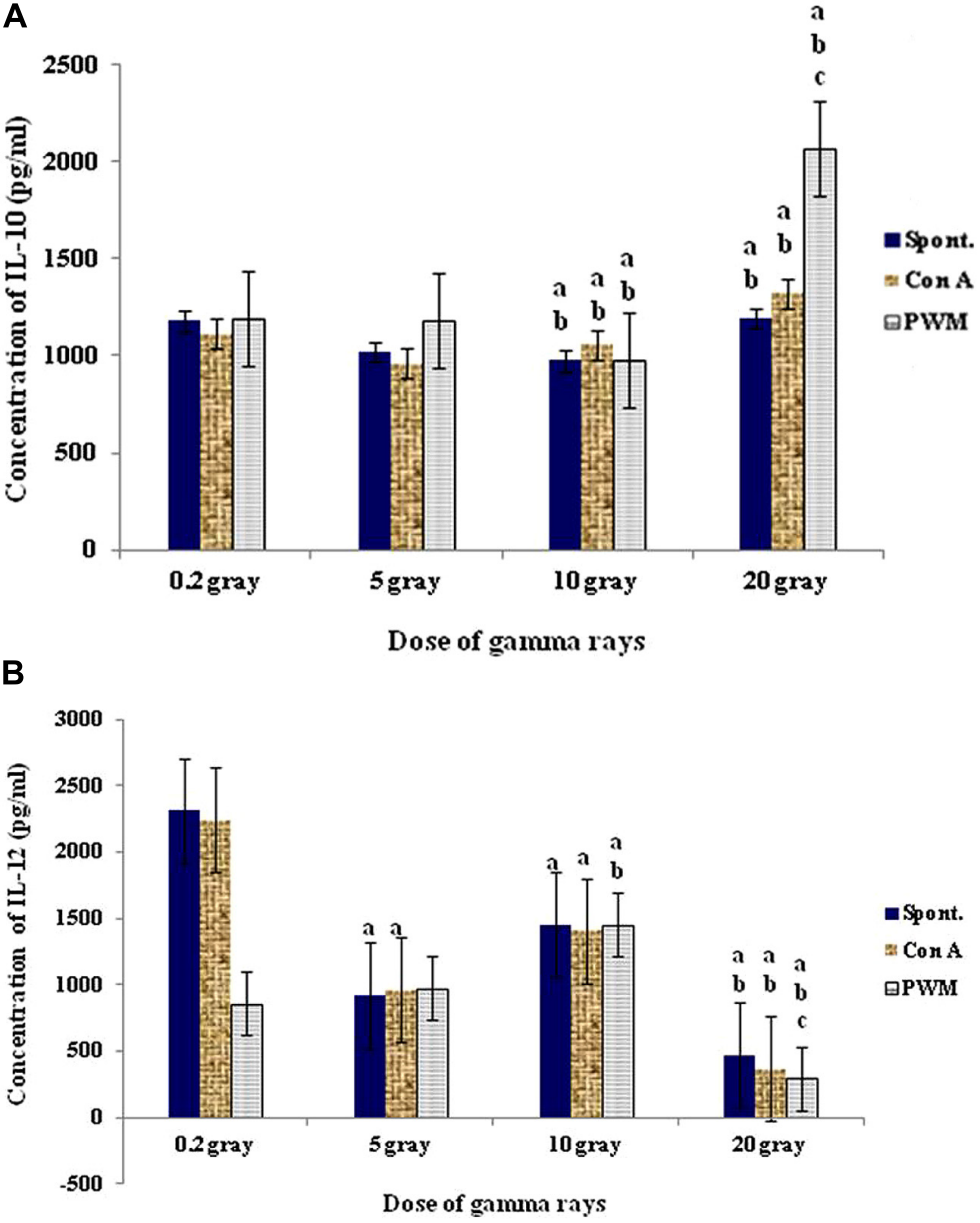
**Concentration of IL-10 (A) and IL-12 (B) release either spontaneously or with *in vitro* stimulation with Con A or PWM after 3 days recovery**. ^a^Level of significance between low doses (0.2 Gy) and higher doses (5,10, 20 Gy) of γ-radiation, ^b^level of significance between 5 gray and higher doses (10, 20 Gy) γ-radiation, ^c^level of significance 10 and 20 Gy γ-rays.

At 3 days recovery, there was a clear cut and dose-dependent tendency for enhanced cell proliferation followed by a sharp decrease at the highest dose (20 Gy). This was associated with a remarkable stimulation of IL-12 production at low dose followed by a gradual suppression at higher doses. IL-10 release was stimulated at low dose (0.2 Gy) then suppressed with doses of 5 and 10 Gy then stimulated with higher doses especially with PWM stimulation (Th2 bias with increasing the dose of γ-rays; **Figure 8**).

**FIGURE 9:**
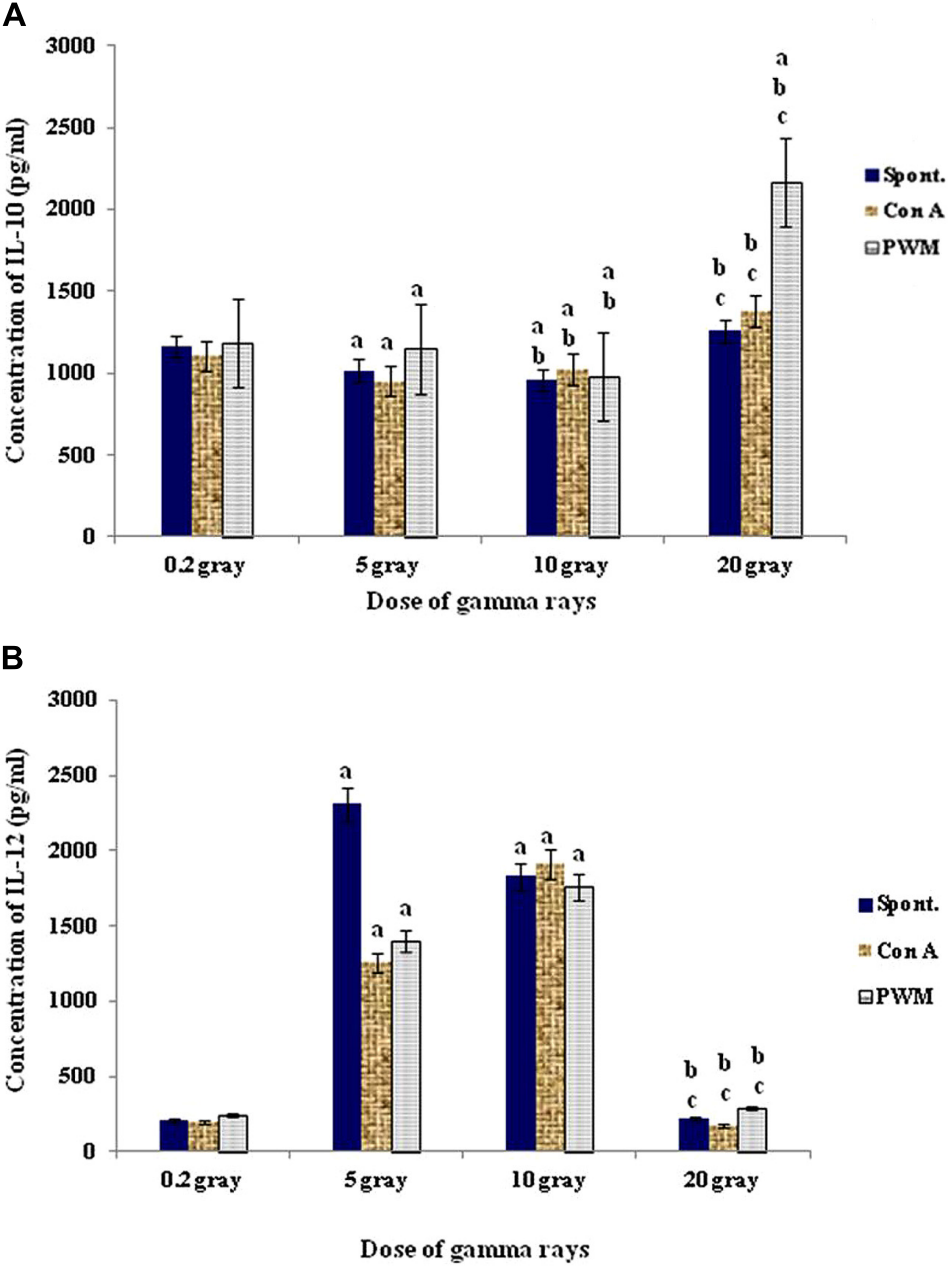
**Concentration of IL-10 (A) and IL-12 (B) release either spontaneously or with *in vitro* stimulation with Con A or PWM after 7 days recovery**. ^a^Level of significance between low doses (0.2 Gy) and higher doses (5,10, 20 Gy) of γ-radiation, ^b^level of significance between 5 gray and higher doses (10, 20 Gy) γ-radiation, ^c^level of significance 10 and 20 Gy γ-rays.

At 7 days recovery, there was a significant dose-dependent enhancement in cell proliferation associated with a remarkable sharp elevation in IL-12 followed by gradual suppression. On the other hand no significant change was recorded in IL-10 release capacity at comparable duration and doses (**Figure 9**).

## Discussion

Radiation is defined as energy emitting from a source, moving through a space and absorbed by an object. It is classified according to the way they interact with normal chemical matter into ionizing and non-ionizing radiation. The ionizing spectrum is divided particulate (alpha, beta, and neutron) and non-particulate (x and γ rays; Shahbakhti et al., 2004). The immunosuppressive effect of gamma irradiation is well-known, but its mechanism has not been examined.

In the immune system, Helper T-lymphocytes induce other immune cells to become better effectors. There are at least two subsets of helper T cells (Th1 and Th2); they secrete very different cytokines upon activation (Sanders, 2006). While the Th1 is characterized by the production of large amounts of IFN-γ, IL-2, TNF-α, and TNF-β to promote cell-mediated immune response, the Th2 cells produces IL-4, IL-10, IL-13, and IL-15 stimulating antibody production (Kidd, 2003). Since Th1 cytokines are important in cellular immunity, the inhibition of its expression by gamma irradiation may contribute to immunosuppressive effects (Han et al., 2002). Different studies indicated that IR causes augmentation of Th2 cytokine production, as IL-4, IL-5, and IL-10 (Han et al., 2002; Noorizadeh et al., 2004). Cellular responses to cytokines may include increased or decreased expression of membrane proteins, secretion of effector molecules and cell proliferation.

However, most studies about the effects of gamma irradiation on cytokine production were carried out in the form of total lymphoid irradiation (TLI) or TBI. In the present study, we examined the effects of different doses of gamma irradiation on lymphoproliferative response in three different situations of splenic cells and cytokine production of splenocytes. We found that exposure of intact spleens to different doses of γ-rays (5, 10, 20 Gy) caused spontaneous and dose-dependent immune stimulation manifested by enhanced cell proliferation and elevated IL-12 production with decreased IL-10 release (i.e., Th1 bias). Variability in the biological response may reflect differences in the total dose, dose rate, end points measured and time of evaluation post-exposure. In this respect, Pecaut et al. (2001) investigated early effects on mice of gamma ray doses of up to 3 Gy at low and high dose rates. They observed a significant dose-dependent loss of spleen and thymus mass after whole-body irradiation, which was somewhat independent of dose rate. They also noticed decreasing numbers of both lymphocytes and leukocytes in the blood and spleen with increasing dose, as well as dose-dependent decreases in lymphocyte subpopulations.

Using the murine model, the functional characteristics of splenocytes and cytokine expression was evaluated after whole body gamma irradiation at various total doses (0.5, 1.5, and 3 Gy) and at low and high dose rates (Gridley et al., 2001). They showed that the basal proliferation of leukocytes in the blood and spleen increased significantly with increasing dose.

Mitogen-induced cell proliferation is employed extensively to assess the general responsiveness of lymphocytes to stimulating agents. Lymphocyte responsiveness to T-cell mitogens [phytohemagglutinin (PHA); concanavalin A; and pokeweed mitogen (PWM)] was shown to depend on experimental conditions. Harrington et al. (1997) showed decreased ability of splenocyte mononuclear cells to respond to the PHA mitogen after whole body gamma irradiation (0–7 Gy). This finding does not correlate with the findings of this work where, direct exposure of splenocytes suspension to 5 Gy of γ-rays revealed significant enhancement of cell proliferation after *in vitro* PWM stimulation. The use of 10 or 20 Gy doses caused significant enhancement in mitogen-induced relative to basal cell proliferation. But there was a significant suppression in spontaneous and mitogen-induced proliferation with increasing γ-rays dose. This may be attributed to a state of conventional immune suppression that is characterized by being dose-dependent and is manifested by decreased cell proliferation and IL-12 release accompanied by increase in IL-10 production (i.e., Th2 bias). In case of the irradiation of intact spleen, mitogens did not enhance the proliferation responsiveness of the cells which correlates with the idea that responsiveness depends on the experimental conditions. In many occasions of our study, ConA was less effective than the PMW mitogen to enhance cellular proliferation. Shankar et al. (2006) studied the bystander effects and adaptive response induced by gamma radiation in murine lymphocytes, using gamma irradiated conditioned medium (γICM) collected from gamma-irradiated lymphocytes. They showed that the ICM enhanced the proliferation of non-irradiated lymphocytes to ConA suggesting that specific soluble factors released by the irradiated lymphocytes trigger signaling pathways that result in increased affinity to ConA mitogen. Unfortunately, these soluble factors have not been identified yet. In our case, we did not use the irradiation medium in the culture.

In addition, we investigated cell proliferation and cytokine production after exposure of murine whole bodies to different doses of γ-rays. We found that exposure of murine whole bodies to low dose γ-rays (0.2 Gy) caused a time-related elevation (at 0 and 3 days recovery) followed by a reduction at 7 days recovery in cell proliferation and IL-12 release. Although there was a pan decrease in IL-10 that was retained at higher levels at day 7. These results indicate a state of immune stimulation following exposure to low dose γ-rays terminated by a remarkable tendency for immune suppression. Mice exposed to 5 or 10 Gy of γ-rays showed time-related enhancement of cell proliferation and IL-12 release while IL-10 release was suppressed indicating again a typical state of immune stimulation (Th1 bias). In contrast, a conclusion that radiation exposure enhanced the Th2 response cytokine production was achieved in murine splenocytes when Bass et al. (1989) used 5.5 and 8.5 Gy of X-rays. When Liu et al. (2003) used low dose whole body irradiated mice with 0.075 Gy X-rays, IL-10 was significantly suppressed and IL-12 mRNA was increased. This behavior was interpreted as a shift of the immune response in favor of Th1 differentiation. At high dose of 20 Gy γ-rays, murine splenocytes showed significant and time-dependent stimulation of cell proliferation associated with significant decrease in IL-12 release and enhancement in IL-10 release indicating that exposure to higher doses of γ-rays caused a shift to Th2 response in splenocytes.

In many systems, an adaptive resistance response to lethality of radiation doses after a challenge acute irradiation is found to be closely related to recovery (Yonezawa et al., 2004). It is well-known that post-irradiation recovery is characterized by gradual reconstitution peripheral blood and bone marrow patterns in rodents and canines as reviewed by Yagunov et al. (1998). When we analyzed the results of the present study as a function of γ-rays dose-dependence at fixed time of recovery, we found that at 0 and 3 days of recovery, there was enhanced cell proliferation and remarkable stimulation of IL-12 production at low dose followed by a gradual suppression at higher doses. This means there is shift to Th2 response with increasing the dose of γ-rays. After 7 days recovery, there was a significant dose-dependent enhancement in cell proliferation associated with a remarkable sharp elevation in IL-12 followed by gradual suppression. On the other hand no significant change was recorded in IL-10 release capacity at comparable duration and doses.

## Conclusion

The immune modulations following γ-irradiation of intact spleen, splenocytes, or whole body are totally different and the use of different doses of IR reflects the difference in radio-sensitivity and/or radio-resistance of immune cells with no correlation between cell proliferation and cytokine production. These finding may have further applications in shifting the immune response to a Th1 type in situations such as tumors or to a Th2 type in autoimmune diseases and parasitic infestation.

## Conflict of Interest Statement

The authors declare that the research was conducted in the absence of any commercial or financial relationships that could be construed as a potential conflict of interest.
